# At the crossroads: Intersectional approaches to women’s health equity

**DOI:** 10.1177/17455057251356890

**Published:** 2025-07-09

**Authors:** Zohra S. Lassi, Jeannette M. Wade, Edward Kwabena Ameyaw

**Affiliations:** 1 Robinson Research Institute, University of Adelaide, SA, Australia; 2 School of Public Health, Faculty of Health and Medical Sciences, University of Adelaide, SA, Australia; 3 Human Health Sciences, University of North Carolina at Greensboro, USA; 4 Institute of Policy Studies and School of Graduate Studies, Lingnan University, Hong Kong, Hong Kong SAR

**Keywords:** women’s health, intersectionality, marginalization

## Abstract

Global shifts in health policies have significantly affected access to reproductive healthcare services, with impacts varying greatly across different populations. Recent policy changes in various regions have included defunding reproductive health programs, promoting abstinence-only education, and restricting international non-government organizations from providing comprehensive reproductive healthcare services. These policies have led to clinic closures, reduced access to maternal and reproductive healthcare in low- and middle-income countries, and increased health inequities. While all low-income individuals may experience varying impacts, the consequences vary dramatically. In the case of undocumented immigrants, insurance options are already severely limited, while specialized care may be inaccessible for people with disabilities. These intersectional realities demonstrate how seemingly uniform policies produce radically different outcomes based on one’s social position. Using the intersectional framework, this article examines how these impacts are magnified for women who experience multiple forms of marginalization and proposes actions for advocates, policymakers, and scholars to safeguard inclusive healthcare access for all women and birthing people.

Recent global health policies have undergone significant changes, affecting healthcare landscapes with disproportionate impacts depending on one’s physical or social location and identities.^1^ An intersectional analysis reveals how policy shifts in reproductive health, educational approaches, and funding allocations create cascading effects that vary greatly depending on race, class, gender identity, sexuality, disability, geographic location, and immigration status.^2^ Notable policy trends include restrictions on international aid, which have prohibited organizations from receiving funding for comprehensive reproductive healthcare services.^3^ These restrictions have led to clinic closures and reduced access to essential reproductive healthcare in low- and middle-income countries (LMICs), which hitherto had relied heavily on external donors.^4^ The immediate effects included service disruptions and increased health inequities, while long-term consequences, such as rising maternal mortality rates, unintended pregnancies, and deepening gender disparities, continue to unfold.^5^ Understanding these impacts through an intersectional lens is crucial for developing inclusive policies that safeguard health access and prevent future setbacks. Here, we highlight the differential impacts of the changing health policies on women and birthing people across the globe, with particular attention to those at the intersections of multiple marginalized identities. We then propose action steps for advocates, interventionists, and scholars to advance health equity through intersectional approaches.

## Intersectional analysis of reproductive health access and education

Intersectionality theory posits that lived experiences are shaped by various aspects of our identities.^2^ So, one is not merely a woman, or middle class, or heterosexual, for example, one is all three, at all times. This means reproductive healthcare must work for women with the fewest resources, not just those with ample resources. It also means that patient–provider interactions must be tailored to meet cultural and religious needs and with consideration for the historical experiences of diverse racial groups.

The impact of restrictive policy approaches on sexual and reproductive health has wide-ranging effects that differ dramatically based on intersecting identities. Accessing adequate healthcare depends on education, availability of services, cultural competence, and financial resources, factors that are distributed unequally across social groups.^6^ People who face multiple axes of oppression encounter unique barriers when seeking to protect themselves from sexually transmitted infections (STIs), conceive, or prevent pregnancy.^7^

Educational approaches that restrict discussions of gender have particularly severe consequences for individuals at the intersections of multiple marginalized identities. Limited education on gender socialization, ideology, and roles affects intimate relationships across all communities, shaping condom negotiations,^8^ sexual partnership dynamics,^9^ and adherence to medical advice.^10^ However, these effects are amplified for young women of color, LGBTQ+ youth, those with disabilities, and those from lower socioeconomic backgrounds who often have fewer alternative resources for accurate information.^11^

Comprehensive sex education, focusing on both abstinence and safe sex, has been shown to equip youth to prevent pregnancy and avoid STIs, with benefits distributed unevenly across social groups.^2^ For example, research demonstrates that comprehensive sex education significantly reduces teen pregnancies, with the largest positive impacts among Black and Latina youth in economically disadvantaged communities.^12^ Conversely, restricting access to education disproportionately harms these same groups, reflecting how policies create different realities for different populations.

The most direct way to ensure reproductive health is through patient care that recognizes and addresses the unique needs of diverse populations. Without access to quality, affordable, and culturally responsive care, women face increased rates of untreated STIs, unintended pregnancies, and maternal and infant mortality.^13^ These outcomes are not distributed equally—Black, Indigenous, and women of color face drastically higher rates of maternal mortality; transgender individuals encounter systemic discrimination in healthcare settings; and rural women often lack geographic access to reproductive health services.^14^ Two critical barriers to care include the lack of cultural sensitivity in healthcare and limited access to affordable insurance options.^15^ When healthcare systems fail to require diversity, equity, and inclusion training, providers lack the necessary skills to effectively serve patients across differences in culture, language, gender identity, sexuality, and disability status.^16^ This particularly affects women at the intersections of multiple marginalized identities who may already face discrimination, language barriers, or historical trauma related to healthcare systems.

Research consistently demonstrates that diversity, equity, and inclusion policies and training are central to creating safe, inclusive spaces and moving closer to health equity.^17^ Similarly, patient–provider concordance, or shared identity factors between patients and healthcare providers, leads to higher quality care and greater patient adherence.^18^ When women can see providers who share aspects of their identities or who have been trained in cultural humility, health outcomes improve.

Regarding healthcare financing, cuts to public insurance programs create cascading effects that differ by social location. While all low-income individuals may lose coverage, the consequences vary dramatically. For undocumented immigrants, insurance options are already severely limited; for people with disabilities, specialized care may become inaccessible; for rural women, the closure of the few available clinics may eliminate all access to care.^19^ These intersectional realities demonstrate how seemingly uniform policies produce radically different outcomes based on one’s social position.

## Call for research through an intersectional lens

The reproductive health of women and birthing people is contingent upon advances in research, biomedical as well as social and behavioral. Traditional research approaches have often failed to account for differences across intersecting identities, producing knowledge that centers dominant groups while marginalizing others. Decades of feminist, intersectional, and critical race scholarship speak to the pitfalls of color-blind, gender-neutral research. These include missing contextual factors that impact marginalized women, reproducing inequality by centering members of dominant groups, and failing to recruit and retain individuals from marginalized populations for clinical trials and other research.

When research funding prioritizes projects centering on intersectionality, researchers can explore how factors like race, gender, sexuality, disability, class, and geographic location interact to produce unique health outcomes and barriers to care.^20^ Such investigations would offer better understanding about the nuances in reproductive health care relative to one’s social position. Without this targeted approach, we cannot create solutions that address the needs of those most marginalized by current systems. Without targeted, solution-focused scholarship that embraces intersectionality as a central analytical framework, the quest to create targeted, solution-focused practice will be a mirage.

## Global health and intersectional justice

The relationship between policy approaches and public health manifests differently across global contexts, with impacts shaped by colonialism, economic exploitation, and geopolitical power dynamics. Health initiatives affecting women must be analyzed through the lens of global power inequities as well as local intersectional realities.

Policies restricting international health funding have profound effects that vary across geographic and social contexts. When organizations that offer comprehensive-reproductive healthcare services lose funding, the effects ripple outward with different implications for different populations.^21^ For example, in regions already experiencing provider shortages, clinic closures may eliminate all healthcare access for rural women. For women living with HIV, reduced funding may interrupt life-saving treatment. For disabled women, specialized services may disappear entirely.

As agencies providing a range of health services to women are compelled to either forgo funding or amend their programs, they face impossible choices about which populations to serve and which services to maintain. Governments and organizations in LMICs that have relied on these funds may be unable to support critical services, particularly for the most marginalized communities. Women who face multiple forms of marginalization—such as those living in poverty, indigenous women, women with disabilities, and women in conflict zones—bear a disproportionate burden of these policy changes.

Resources for HIV prevention, family planning, and maternal health services have been severely constrained by funding cuts aimed at initiatives like the United Nations Population Fund (UNFPA). In places like South Asia and Africa, where public health systems are fragile due to histories of colonialism and economic exploitation, these limitations have had particularly severe adverse effects.^22^ However, these effects are not uniform across or within regions. Urban women may retain some access to services while rural women lose all access; women from dominant ethnic groups may find alternative resources while indigenous women face complete service gaps; and wealthy women may travel to access care while poor women have no options.

The intersection of global health funding with local realities creates complex patterns of access and exclusion. Withdrawing funding not only jeopardizes current health programs but also inhibits creative solutions to the particular health issues that women in these areas face.^4^ Trans women, women of color, and women living in rural areas often face compounded challenges accessing care under these conditions, showing how global policies interact with local power structures to produce unique patterns of health inequity.

## Multiple health crises and intersectional vulnerability

The closure of HIV and contraception programs has created overlapping health crises with differential impacts based on social location. Increased obstacles to accessing reproductive health services have resulted from the termination of funding for initiatives that offered education and access to contraceptives. The decrease in financing for HIV prevention programs, such as those that provide antiretroviral therapy and education, has put women’s health at higher risk in areas with high HIV prevalence, such as sub-Saharan Africa.^23^

These overlapping crises create a precarious situation where women, particularly those at the intersections of multiple marginalized identities, face compounded vulnerability. For example, a young woman who is HIV-positive, living in a rural area, and from an indigenous community may lose access to HIV treatment, contraception, and maternal healthcare simultaneously, creating a level of vulnerability far greater than any single factor would suggest. This demonstrates the importance of intersectional analysis in understanding how health policies create radically different realities for different populations.

## Mental health through an intersectional lens

Policy changes affecting reproductive healthcare access have alarming implications for mental health that vary across social groups. Due to limited access to contraception, unwanted pregnancies increase, posing serious psychological as well as physical and socioeconomic challenges. The decreased accessibility of sexual health services contributes to anxiety around STIs and pregnancy. The stigma around abortion and the psychological anguish of unwanted pregnancies combine to create complex mental health challenges that manifest differently across cultural contexts and social positions.

The negative effects of these policy changes may be exacerbated for women who are unable to obtain essential health services, raising their risk of anxiety, depression, and other mental health issues. Women who are already marginalized due to factors such as race, ethnicity, sexual orientation, gender identity, disability, or socioeconomic status experience these burdens most acutely, often without access to culturally appropriate mental health support. This demonstrates how reproductive health policies create cascading effects across multiple dimensions of wellbeing, with patterns that can only be understood through intersectional analysis.

## Resistance through intersectional solidarity

Restrictive-reproductive health policies have sparked widespread resistance that increasingly embraces intersectional approaches. Grassroots movements, non-government organizations (NGOs), and international organizations have played critical roles in mitigating adverse policy effects, advocating for inclusive change, and ensuring continued access to essential healthcare services while centering those most marginalized.

Grassroots movements have been at the forefront of resistance, mobilizing communities, raising awareness, and demanding action from policymakers. Organizations led by Black, Indigenous, and women of color have challenged restrictive-reproductive health policies while addressing how these policies create unique barriers for different communities.^24^ The reproductive justice framework, developed by women of color activists, explicitly connects reproductive rights to social justice, recognizing that the ability to have or not have children, and to parent those children in safe environments, is shaped by multiple systems of oppression.^25^

In LMICs, local feminist organizations with intersectional approaches have worked to fill service gaps left by funding cuts, ensuring that women and marginalized groups continue to access sexual and reproductive healthcare.^26^ These organizations often develop innovative approaches that address local needs while challenging global power inequities, demonstrating how resistance must be contextual as well as intersectional.

Digital activism has become a powerful tool in resisting restrictive policies across national boundaries. Campaigns highlighting reproductive justice have amplified diverse voices globally, drawing attention to how policies affect different populations in different ways. By leveraging social media, grassroots movements have raised funds, connected activists across borders, and pressured governments to prioritize women’s health with attention to intersecting forms of oppression.^27^

These digital networks have facilitated new forms of transnational solidarity that recognize both shared struggles and contextual differences. For example, activists from different regions may share strategies for delivering healthcare in restrictive environments while acknowledging how local factors shape which approaches will be effective in different contexts. This transnational solidarity demonstrates how resistance to reproductive health restrictions increasingly embraces both global connection and contextual specificity.

NGOs and international bodies have been instrumental in developing alternative approaches to reproductive healthcare in restrictive environments. Organizations like the International Planned Parenthood Federation (IPPF) and Marie Stopes International have redirected funding and strengthened partnerships to maintain reproductive health services despite funding withdrawals, with increasing attention to serving women with multiple marginalized identities.^28^

Several governments, particularly in Europe and Canada, have launched funding initiatives such as the SheDecides Movement, which mobilizes resources to counteract restrictive policies.^29^ These collective efforts highlight the resilience of global advocacy networks and the necessity of sustained resistance to policies that threaten women’s health worldwide, particularly for those most vulnerable due to intersecting forms of marginalization.

## Future directions for intersectional health justice

Limited funding for comprehensive reproductive healthcare has led to service gaps that disproportionately affect the most marginalized communities. Alternative funding models must be developed that prioritize the needs of those at the intersections of multiple oppressions. Scholars are urged to track and contextualize the impact of funding restrictions through an intersectional lens, conduct public opinion research to understand the stance of global citizens on these policies, and evaluate projects that meet the sexual and reproductive health needs of diverse populations.

Developing sustainable funding streams that are less vulnerable to political shifts requires innovative approaches at the local, national, and international levels. Community-based funding models, social enterprise approaches, and multi-lateral funding collaborations all offer potential alternatives that may provide more stable support for comprehensive reproductive healthcare. These approaches must center the leadership and perspectives of communities most affected by funding restrictions.

As gender becomes increasingly politicized, research and practice must embrace the full complexity of gender identities, expressions, and experiences. Trans and non-binary people face particular barriers to reproductive healthcare, while cisgender women across other social locations encounter gender-specific obstacles shaped by race, class, disability, and other factors. Scholars are urged to use intersectional, gendered, holistic and queer theories to highlight the connections between health and gender while acknowledging differences across other identity dimensions.

Advancing gender-inclusive reproductive healthcare requires challenging binary assumptions, developing new language and frameworks, and creating services that recognize diverse gender experiences. This work must center the leadership of trans and non-binary communities while also addressing how gender interacts with other systems of power and oppression to shape reproductive health access and outcomes.

Traditional research approaches have often reproduced colonial power dynamics and centered Western perspectives while marginalizing indigenous knowledge and community wisdom. Decolonizing research on reproductive health requires challenging these hierarchies and developing methodologies that center marginalized communities as knowledge producers rather than research subjects.

Work on women’s health specifically must acknowledge the historical exploitation of women of color in medical research and practice, from gynecological experiments on enslaved women to forced sterilization of indigenous women to contemporary racial disparities in maternal mortality. Addressing these historical and ongoing injustices requires research approaches that explicitly challenge medical racism and colonialism while developing new, community-centered models of knowledge production.

Scholars, grant writers, and other advocates are challenged to seek out innovative means to fund intersectional, decolonial research and to share those discoveries with the community at large. Given the global nature of reproductive health challenges, we cannot operate in silos but must build collaborative approaches that respect both contextual differences and shared struggles.

## Conclusion

The complex intersections between policy decisions, social location, and public health create dramatically different realities for women and birthing people across the globe. Policies restrictive-reproductive healthcare access have worked against advancements in women’s health, especially in already marginalized communities and regions. Women who face multiple forms of oppression—based on race, class, disability, sexuality, gender identity, and geographic location—experience unique patterns of vulnerability that can be best understood through intersectional analysis. Advancing reproductive health justice requires challenging restrictive policies while building alternative models that center those most marginalized. This entails supporting comprehensive health services that address the specific needs of diverse communities, developing innovative funding approaches that are less vulnerable to political shifts, and centering the leadership of those most affected by current inequities. Ultimately, protecting women’s health through an intersectional lens is crucial for the well-being and prosperity of entire societies, considering women’s critical role in society and nation building. By guaranteeing that every person, regardless of their social location, has access to the healthcare services they require, we can build healthier communities and support global sustainable development. Recognizing reproductive health as a basic human right and committing to equity and justice through intersectional approaches must be the guiding principles for future policy, practice, and research in women’s health globally.

## References

[1] Tanne JH. Trump presidency will mean changes for healthcare, reproductive rights, and global heating. British Medical Journal Publishing Group, 2024. 10.1136/bmj.q2461 39510559

[2] Bowleg L. The problem with the phrase women and minorities: intersectionality—an important theoretical framework for public health. Am J Public Health 2012; 102(7): 1267–1273. 22594719 10.2105/AJPH.2012.300750 PMC3477987

[3] Sherman C. Trump signs order to reinstate ‘global gag rule’ on abortion aid. The Guardian, 2025.

[4] Ahmed Z. The Unprecedented Expansion of the Global Gag Rule: Trampling Rights, Health and Free Speech. The Guttmacher Institute, https://www.guttmacher.org/gpr/2020/04/unprecedented-expansion-global-gag-rule-trampling-rights-health-and-free-speech (2020).

[5] Institute G. Abortion Worldwide 2019: Uneven Progress and Unequal Access. Guttmacher Institute, https://www.guttmacher.org/report/abortion-worldwide-2019 (2019).

[6] Nunn J Freeman R Anderson E , et al. Inequalities in access to education and healthcare. Eur J Dent Educ 2008; 12: 30–39. 18289266 10.1111/j.1600-0579.2007.00478.x

[7] Henriques E Schmidt C Pascoe R , et al. Counter-narratives of structural oppressions, stigma and resistance, and reproductive and sexual health among youth experiencing homelessness. Qual Health Res 2022; 32(10): 1447–1463. 35739061 10.1177/10497323221110694 PMC9411701

[8] Woolf SE Maisto SA. Gender differences in condom use behavior? The role of power and partner-type. Sex Roles 2008; 58: 689–701.

[9] Rogowska A Tofel M Zmaczyńska-Witek B , et al. The relationship of number of sexual partners with personality traits, age, gender and sexual identification. Psychol Sex 2022; 13(2): 147–164.

[10] Babitsch B Bormann C Gohl D , et al. Gender and utilization of health care. In: Janssen C, Swart E and von Lengerke T (eds) Health care utilization in Germany: theory, methodology, and results. 2014, Springer, pp. 101–116.

[11] Gower AL Rider GN del Río-González AM , et al. Application of an intersectional lens to bias-based bullying among LGBTQ+ youth of color in the United States. Stigma Health 2023; 8(3): 363. 37936868 10.1037/sah0000415 PMC10627550

[12] Stanger-Hall KF Hall DW. Abstinence-only education and teen pregnancy rates: why we need comprehensive sex education in the US. PLoS One 2011; 6(10): e24658. 10.1371/journal.pone.0024658 PMC3194801 22022362

[13] Malarcher S Olson L Hearst N. Unintended pregnancy and pregnancy outcome: equity and social determinants. Equity, social determinants and public health programmes. 2010, World Health Organization, pp. 177–197.

[14] Bridges KM. Racial disparities in maternal mortality. NYUL Rev 2020; 95: 1229.

[15] Call KT McAlpine DD Garcia CM , et al. Barriers to care in an ethnically diverse publicly insured population: is health care reform enough? Med Care 2014; 52(8): 720–727. 25023917 10.1097/MLR.0000000000000172

[16] Betancourt JR Corbett J Bondaryk MR . Addressing disparities and achieving equity: cultural competence, ethics, and health-care transformation. Chest 2014; 145(1): 143–148. 24394825 10.1378/chest.13-0634

[17] Stanford FC. The importance of diversity and inclusion in the healthcare workforce. J Natl Med Assoc 2020; 112(3): 247–249. 32336480 10.1016/j.jnma.2020.03.014 PMC7387183

[18] Shen MJ Peterson EB Costas-Muñiz R , et al. The effects of race and racial concordance on patient-physician communication: a systematic review of the literature. J Racial Ethn Health Disparities 2018; 5: 117–140. 28275996 10.1007/s40615-017-0350-4 PMC5591056

[19] Hacker K Anies M Folb BL , et al. Barriers to health care for undocumented immigrants: a literature review. Risk Manag Healthc Policy 2015; 8: 175–183. 26586971 10.2147/RMHP.S70173 PMC4634824

[20] Nakkeeran N Nakkeeran B. Disability, mental health, sexual orientation and gender identity: understanding health inequity through experience and difference. Health Res Policy Syst 2018; 16(Suppl 1): 97. 30301453 10.1186/s12961-018-0366-1 PMC6178246

[21] Hewitt S McNiesh S Fink L . Barriers to primary care access in rural medically underserved areas: immediate care: a simple solution to a complex problem. Online J Rural Nurs Health Care 2019: 127–155.

[22] Abd Rahim A Abdul Manaf R Juni MH , et al. Health system governance for the integration of mental health services into primary health care in the sub-Saharan Africa and South Asia region: a systematic review. Inquiry 2021; 58: 00469580211028579. 10.1177/00469580211028579 PMC8293855 34275346

[23] Hailu BA. Trend and principal components of HIV/AIDS among adults in SSA. Sci Rep 2024; 14(1): 11098. 38750039 10.1038/s41598-024-55872-2 PMC11096374

[24] Women’s March. Reproductive rights and resistance: a movement for justice, https://www.womensmarch.com (2021).

[25] Morison T. Reproductive justice: a radical framework for researching sexual and reproductive issues in psychology. Soc Personal Psychol Compass 2021; 15(6): e12605.

[26] Mavodza C Goldman R Cooper B. The impacts of the global gag rule on global health: a scoping review. Glob Health Res Policy 2019; 4: 1–21. 10.1186/s41256-019-0113-3 PMC6714436 31485484

[27] Gantt-Shafer J. “They Just Went After Us:” reproductive justice advocacy at an abortion fund. Front Commun 2020; 5: 501276.

[28] International Planned Parenthood Federation. How we are countering global funding gaps, https://www.ippf.org (2022).

[29] SheDecides. Global action for women’s health: a response to policy rollbacks, https://www.shedecides.com (2023).

